# Pleistocene climate fluctuations as the major driver of genetic diversity and distribution patterns of the Caspian green lizard, *Lacerta strigata* Eichwald, 1831

**DOI:** 10.1002/ece3.7543

**Published:** 2021-05-02

**Authors:** Reihaneh Saberi‐Pirooz, Hassan Rajabi‐Maham, Faraham Ahmadzadeh, Bahram H. Kiabi, Mohammad Javidkar, Miguel A. Carretero

**Affiliations:** ^1^ Department of Biodiversity and Ecosystem Management Environmental Sciences Research Institute Shahid Beheshti University Tehran Iran; ^2^ Animal Sciences and Marine Biology Faculty Life Sciences and Biotechnology Shahid Beheshti University Tehran Iran; ^3^ School of Biological Sciences the University of Adelaide Adelaide SA Australia; ^4^ CIBIO Research Centre in Biodiversity and Genetic Resources InBIO Universidade do Porto Porto Portugal; ^5^ Departamento de Biologia Faculdade de Ciências da Universidade do Porto Porto Portugal

**Keywords:** Alborz Mountains, glaciation, Hyrcanian Forests, *Lacerta strigata*, Pleistocene, refugia

## Abstract

Green lizards of the genus *Lacerta* have served as excellent models for studying the impact of Pleistocene climatic oscillations on genetic structures. The Caspian green lizard, *Lacerta strigata*, occupies various habitats across the Caucasus and the South Caspian Sea, with the Hyrcanian Forests and north of the Alborz Mountains forming the core of the range. This study aimed to re‐examine the phylogenetic relationships of *L. strigata* with other congeneric members and to assess the genetic structure and historical demography of the species. Furthermore, Species Distribution Models (SDMs) were performed to infer the species' potential habitat suitability and were then projected on climate scenarios reflecting current and past (6 ky and 21 ky before present) conditions. A total of 39 individuals collected from most of the distribution range, together with additional lacertid species sequences from the GenBank database, were examined using mtDNA (Cyt *b* and 12S ribosomal RNA) and nuclear (C‐mos and β‐fibrinogen) sequence data. Based on the phylogenetic analyses, *L. strigata* was found to be a sister taxon to all other members of the genus. The species included two main clades (regional western and eastern) that diverged in a period between the Early and Middle Pleistocene. Based on the BBM and S‐Diva analyses, both dispersal and vicariance events explained the phylogeographic structure of the species in the Hyrcanian Forests. The historical demographic analyses using Bayesian skyline plots showed a mild increase in the effective population size from about 120 Kya for the western regional clade. According to phylogeographic structures and SDMs evidence, as in other species within the region, it appears that the south of the Caspian Sea (Hyrcanian Forests), and the Alborz Mountains acted as multiple refugia during cold periods and promoted expansion outwards amid the warm periods. Overall, the results provided evidence that the genetic structure of the species has been influenced by the Pleistocene climatic fluctuations.

## Graphical abstract

The Caspian green lizard, *Lacerta strigata*, occupies various habitats across the Caucasus and the South Caspian Sea, with the Hyrcanian Forests and north of the Alborz Mountains forming the core of the range. The regions acted as multiple refugia during cold periods and promoted expansion outwards amid the warm periods. Overall, the results provided evidence that the genetic structure of the species has been influenced by the Pleistocene climatic fluctuations.

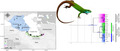

## 1 INTRODUCTION

Pleistocene climatic fluctuations are determined by intervals of cold and warm cycles during glacial and interglacial periods (Cilek & Smith, 2009; Davis, 1981; Ehlers & Gibbard, 2007; Hewitt, 2000; Petit et al., 2005; Svenning et al., 2015). In addition to the key role of refugia in species survival during the glacial periods, nowadays, they are also imperative for conservation due to their climate stability in contrast to fluctuating environments in the remaining parts of the species ranges (e.g., Sillero & Carretero, 2013). Glacial refugia have had a major influence on current patterns of genetic diversity and distribution of species (Avise, 2000; Hewitt, 2004; Himes et al., 2008; Taberlet et al., 1998). Whereas the classic research on the impact of the Pleistocene on phylogeographic patterns focused on southern European Peninsulas (Gómez & Lunt, 2007), the latest investigations have uncovered comparable refugia dynamics in the Caucasus and the Middle East (Gvoždik et al., 2010; Tarkhnishvili, 2014). These refugial areas include the east and southeast of the Black Sea (Rato et al., 2021; Tuniyev, 2011), the east and south of Lake Van (Albayrak et al., 2012; Dubey et al., 2006; Médail & Diadema, 2009), the southeastern slopes of the Greater Caucasus and the eastern Lesser Caucasus (Gabelaia et al., 2015; Van Andel & Tzedakis, 1996), the Zagros Mountains, the Alborz Mountains, and the southern coast of the Caspian Sea in Iran (Ahmadzadeh, Flecks, Carretero, Mozaffari, Böhme, et al., 2013; Ahmadzadeh, Flecks, Rödder, et al., 2013; Dianat et al., 2017). In particular, the three latter seem to have harbored several animal and plant species during the glaciations (Ashrafzadeh et al., 2016; Dufresnes et al., 2016; Leroy & Arpe, 2007; Naderi et al., 2014; Naqinezhad et al., 2008; Ramezani et al., 2008; Saberi‐Pirooz et al., 2018; Tarkhnishvili et al., 2011; Tóth et al., 2013; Veith et al., 2003; Zohary, 1973).

Lacertid lizards represent excellent models to study the speciation and impacts of climate fluctuations on genetic structure (Ahmadzadeh et al., 2013). In most Palaearctic lacertids speciation that took place before the Pleistocene, the glacial periods seem to be responsible for shaping intraspecies structures (e.g., Ahmadzadeh, Flecks, Rödder, et al., 2013; Barata et al., 2012; Joger et al., 2007). In particular, oriental green lizards are widely distributed from western Europe to central Asia and occur in various environmental conditions (Godinho et al., 2005). The Caspian green lizard (*Lacerta strigata*) is one of nine well‐known species of the genus *Lacerta* (Arnold et al., 2007; Kornilios et al., 2020) distributed from the northeastern/central Caucasus, and the northeastern Anatolia to the southern Caspian coast (including Russia, Armenia, Azerbaijan, Georgia, Turkey, Iran, and Turkmenistan) (Tuniyev et al., 2009). It occupies a variety of habitats, from clay semideserts and steppes to shrublands and from lowlands up to 3,000 m a. s. l. in the southern Caspian Sea (Anderson, 1999). In Iran, *L. strigata* is found in the Alborz mountain range, which extends along the southern coast of the Caspian Sea from Talysh (Iran, Azerbaijan) in the west to Kopeh Dag Mountains in (Allen et al., 2003; Ghorbani, 2013; Stocklin, 1968). This mountain range is regarded as a biogeographic barrier for fauna and flora exchange between the southern and northern sides (Ghorbani, 2013; Mozaffarian, 2013).

In the south of the Caspian Sea, the species is mainly found in humid habitats of the Hyrcanian Forests (Ahmadzadeh et al., 2008; Anderson, 1999; Langerwerf, 1980; Šmid et al., 2014), which forms a long and narrow vegetation belt on the northward slopes of the Alborz Mountains (Naqinezhad et al., 2012; Siadati et al., 2010). These forests, acting as a center of cryptic and endemic diversities, are regarded as an ancient ecosystem that has provided refuge for many species. It is generally considered that the climatic stability during the Pleistocene has played a significant role in shaping the current rich Hyrcanian fauna. However, few works have investigated biogeographical processes that contributed to the present diversity (see Ahmadi et al., 2018; Ahmadzadeh, Flecks, Rödder, et al., 2013; Leestmans, 2005;  Ramezani et al., 2008).

In contrast to other congeneric species, the ecology and evolutionary history of the Caspian green lizard remain poorly investigated (Kafash et al., 2019). Besides, the phylogenetic position of the species within the genus is not well determined (Ahmadzadeh, Flecks, Rödder, et al., 2013; Godinho et al., 2005). Therefore, this study aims to determine the phylogenetic position of *L. strigata* within the green lizards, to assess the phylogeographic patterns of the species in the context of the Pleistocene climatic oscillations, and to identify biogeographic processes that shaped the genetic structure across its distribution range using a multilocus approach including mitochondrial (Cyt *b* and 12S) and nuclear (C‐mos and β‐fib) genes combined with species distribution models (SDMs).

## 2 MATERIAL AND METHODS

### 2.1 Sample collection

**FIGURE 1:**
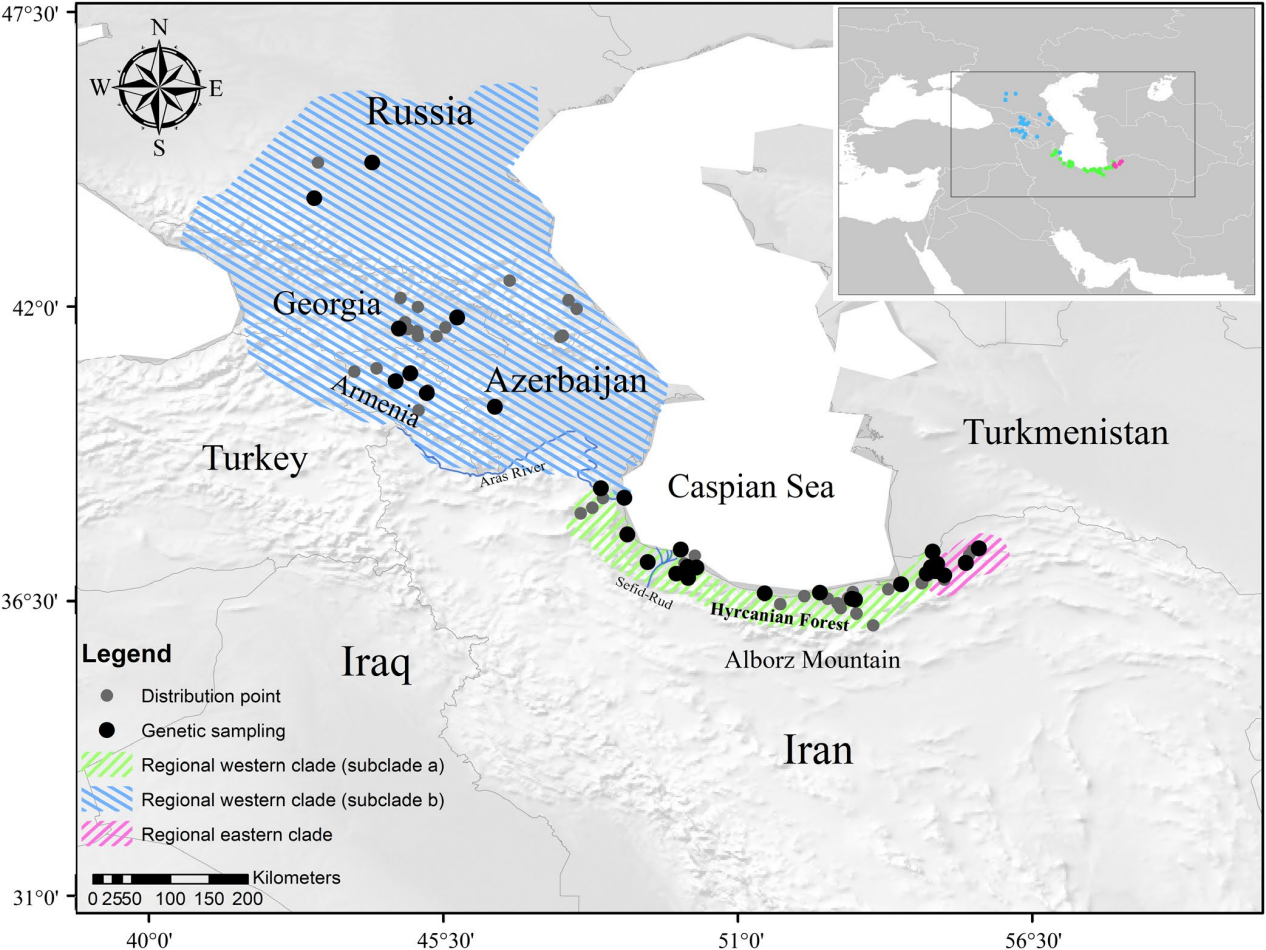
The distribution range of *Lacerta strigata*. The gray circles represent distribution points (the points were obtained in this study), and black circles refer to the locations of samples used in the genetic analyses. The specimens of the regional eastern clade, the regional western clade with two subclades “a” and “b” marked with pink, green, and blue crosshatches, respectively

We collected a total of 39 specimens of the Caspian green lizard from most of its distribution range but mainly focused on the putative habitats within the southern coast of the Caspian Sea (Table S1 and Figure 1). A small part of the tail tip was removed and specimens were released in the site of capture. Tissue samples were preserved in 96% ethanol and stored in a freezer at −20℃ for long‐term maintenance.

### 2.2 Laboratory procedures

Total genomic DNA was extracted with standard protocols of high‐salt and phenol‐chloroform methods (Sambrook et al., 1989). Four partial genes including two mtDNA (Cytochrome b (Cyt *b*) and 12S ribosomal RNA (12S)) and two nuclear (Oocyte maturation factor Mos (C‐mos) and β‐fibrinogen (β‐fib)) markers were used. Primer pairs used for Cyt *b* and 12S included GluDg/Peil (Engström et al., 2007; Palumbi, 1991) and 12Sa/12Sb (Kocher et al., 1989), respectively. The nuclear primer pairs were L1zmos and Hcmos1 for C‐mos (Pavlicev & Mayer, 2006) and FIB‐BI7U and FIB‐BI7L (Prychitko & Moore, 1997) for β‐fib. PCRs were performed in a total volume of 25 µl containing 12.5 µl of Master Mix Red (Ampliqon, Copenhagen, Denmark), 0.5 µl of each primer, 10.5 µl dd $H_{2}O$, and 1 µl of template DNA (50–100 ng). PCRs were carried out separately for each gene under the conditions that are mentioned in Godinho et al. (2005) and Ahmadzadeh et al. (2012). PCR products were visualized on 1% agarose gel. The successfully amplified PCR samples were then sent to Macrogen (Macrogen, Seoul, South Korea) for sequencing. Sequences were edited using CodonCode Aligner v.6.0.2.X program (CodonCode Corporation, Dedham, MA, USA). The generated sequences were submitted to the GenBank database (Table S1).

### 2.3 Alignments and phylogenetic analyses

To determine the phylogenetic position of *L. strigata* among other green lizards, additional sequences from other species of Lacertidae retrieved from Ahmadzadeh et al. (2013) were added (see Table S2) to the generated sequence dataset. The datasets of all genes were aligned with MAFFT v.6 (Katoh et al., 2017) (https://mafft.cbrc.jp/; algorithm: Auto; scoring matrix: 200Pam/k = 2; Gap open penalty: 1.53) and were then combined, resulting in a final 2,178 bp alignment (Cyt *b*: 846 bp, 12S: 355 bp, C‐mos: 514 bp, and Β‐fib: 463 bp).

The best‐fit nucleotide substitution models were obtained for each gene under the Akaike's information criterion (Akaike, 1974) using MrModeltest v.2.3 (Nylander, 2004). As a result, the following models were selected: Cyt *b*: HKY + I + G (Hasegawa et al., 1985; Yang, 1996; I = 0.5459, G = 2.0188); 12S: GTR + I + G (Rodriguez et al., 1990; Yang, 1996; I = 0.5910, G = 0.9886); and C‐mos and β‐fib: HKY + G (Hasegawa et al., 1985; Yang, 1996; G = 0.7556 and G = 1.0111, respectively). The Bayesian Inference (BI) analysis was conducted using MrBayes v.3.2 (Huelsenbeck & Ronquist, 2001). The analysis was performed using two independent and simultaneous runs (four chains for each run) with 10^7^ generations. Subsampling trees and parameters were saved every 100th generation, which produced 10^5^ trees during the analysis. Finally, 10% of trees were discarded as burn‐in, and the remaining trees (including 10,001 trees) were used to reconstruct the 50% majority‐rule consensus tree. The final standard deviation (*SD*) of split frequencies for the combined dataset (four genes) was 0.0013. The parameters were separately calculated for each gene partition. The performance of each run and assessment of convergence were thereafter explored using Tracer v.1.6 (Rambaut & Drummond, 2009). The Maximum Likelihood (ML) analysis was carried out using RAxML v.8.2.X (Stamatakis, 2016) under the GTR + G + I model for each gene partition using 1,000 bootstrap pseudoreplicates to assess the confidence of branches. Uncorrected genetic distances were calculated with PAUP v.4.0a10 (Swofford, 2003) for Cyt *b* sequences.

### 2.4 Estimation of divergence times

Divergence times were estimated with BEAST v.1.7.2 (Drummond & Rambaut, 2007) using the combined dataset (Dataset 1: including 29 species of Lacertidae). To calibrate the analysis, two fossil records and one geological event were used: *Lacerta ruscinensis* (5.3 million years ago (henceforth Mya), used as the minimum age of the European *Timon* since it is morphologically similar to the extant European *Timon* spp. (Estes, 1983) and *Lacerta* sp. (17.5 Mya, used as the minimum age of *Lacerta* since it is morphologically different from *Timon*, but shared some common features of *Lacerta* spp., Červnansky, 2010). The split between the Canarian lacertids *Gallotia caesaris caesaris* and *Gallotia caesaris gomerae* due to the formation of El Hierro Island (1.05 Mya, Guillou et al., 1996; Carranza et al., 2004) was used for calibration. The abovementioned calibration points were applied to the *Timon‐Lacerta* node (gamma distribution, shape: 1, scale: 0.5; 95% CI: 17.51–19.34 Mya), the most recent common ancestral (henceforth MRCA) node of *T. lepidus* and *T. pater* (gamma distribution, shape: 1, scale: 0.2; 95% CI: 5.30–6.03 Mya), and the MRCA node of *Gallotia caesaris caesaris* and *Gallotia caesaris gomerae* (normal distribution, M: 1.05, S: 0.02; 95% CI: 1.01–1.09 Mya). A lognormal relaxed clock (uncorrelated) was used for all markers with the Yule model for the speciation prior. The analysis was run for 2 × 10^7^ generations and sampling every 10^3^ generations. The mutation rates were estimated for each gene, and the sequence dataset of *L. strigata* (Dataset 2: including 15 individuals of *L. strigata*) was calibrated under the calculated mutation rates (Cyt *b*: 1.47 × 10^–2^, 12S: 4.7 × 10^–3^, C‐mos: 7.64 × 10^–4^ and β‐fib:1.98 × 10^–3^ substitutions per site per million years; see Results). The analysis using the combined dataset was performed under the coalescent approach with a lognormal relaxed clock model (uncorrelated). A maximum clade credibility tree was reconstructed using the MCMC analyses for two independent runs of 20 million generations, sampling every 1,000 generations. Convergence diagnostics for the MCMC analyses were assessed using Tracer v. 1.6.1.

### 2.5 Population structure

An analysis of molecular variance (AMOVA) was performed to examine the population status within *L. strigata* based on the Cyt *b* marker for 39 individuals. Since the phylogenetic tree revealed two distinct clades (see Results), each was considered as a single population. The AMOVA test and the standardized measure of genetic differentiation (F*st*) were calculated using Arlequin v.3.5 (Excoffier & Lischer, 2010) with 10,000 permutations.

### 2.6 Demographic analysis

Molecular diversity indices including the number of haplotypes (H), haplotype diversity (*h*), nucleotide diversity (*π*), and the number of polymorphic sites (S) were estimated for the regional clades based on Cyt *b*. Demographic history analyses, that is, Tajima's *D* (Tajima, 1989) and Fu's *fs* (Fu, 1997) indices were calculated with Arlequin v.3.5.

To estimate the frequency distribution of the pairwise nucleotide differences, a Mismatch Distribution (MMD) analysis was separately performed for each population, assuming a sudden expansion with spatial parameters.

To investigate variations in the effective population size (henceforth *N*_e_) against time for *L. strigata*, the Bayesian skyline plot (BSP; Drummond et al., 2005) was constructed using the Cyt *b* gene just for the western regional clade because of adequate available samples (*n* = 29). The BSP was performed with BEAST v1.7.2 under the strict clock at the calculated rate of 1.47 × 10^–2^ per site per Mya. The analysis was done for 5 × 10^6^ generations with log parameters sampled every 100 iterations.

### 2.7 Biogeographic analysis

For reconstructing the possible ancestral range of *L. strigata*, the statistical dispersal vicariance (S‐DIVA) and Bayesian binary MCMC (BBM) analyses were executed using RASP 2.1 beta (Yu et al., 2015) for Cyt *b* because of the adequate available samples and comparatively higher mutation rate. Three different areas considered within its distribution range included the eastern (E) and central (C) regions of the Hyrcanian Forests in Iran, and the western (W) part representing the Astara samples (west of the forests) and most of Iran's northwestern territories (see Figure 1). To take into account phylogenetic uncertainty, 20,000 trees generated from the Mr Bayes tree were set as the input file for S‐DIVA. The BBM analysis was run for 5 × 10^6^ generations under ten MCMC, and the sampling frequency was every 100 generations. The fixed Jukes–Cantor model with equal among‐site rate variation was used for the BBM analysis.

The parsimony haplotype networks were drawn with TCS v.1.21 (Clement et al., 2000) for Cyt *b* under 95% probability.

### 2.8 Species distribution modeling (past–present)

#### 2.8.1 Species occurrence points

The species occurrence localities were compiled from our fieldwork, museum collections, publications, and the global biodiversity information facility (www.gbif.org). Reliability of all records was assessed by mapping them in DIVA‐GIS 7.4 (Hijmans et al., 2005, available through http://www.divagis.org). In total, 139 unique records were considered for model building covering the whole known geographic range of the species (Table S4).

#### 2.8.2 Climate data and variable selection

The 19 so‐called BIOCLIM variables with a grid cell resolution of 2.5 arc.min were obtained from the second version of the WorldClim (version 2.1) database which represents historical monthly weather data as the averages of the period 1970–2000 (Fick & Hijmans, 2017; http://www.worldclim.org/bioclim). To reduce the negative effects of multicollinearity of predictors, we used a subset of independent variables. The selection was based on the species ecological requirements and a pairwise correlation matrix, using a Pearson's r score threshold of 0.75. As such, for the SDM computation, we conserved as predictors only seven variables with *R* ^2^ < 0.75. The final subset of variables included: BIO1 = Annual Mean Temperature; BIO2 = Mean Diurnal Range; BIO5 = Max Temperature of Warmest Month; BIO8 = Mean Temperature of Wettest Quarter; BIO12 = Annual Precipitation; BIO13 = Precipitation of Wettest Month; BIO15 = Precipitation Seasonality.

To reconstruct the species historical habitat suitability during the Mid‐Holocene (~6ky BP) and Last Glacial Maximum (LGM; ~21ky BP), we used unweighted ensembles based on palaeoclimate simulations following the r1i1p1 ensemble from the PMIP3 project (Paleoclimate Modeling Intercomparison Project Phase III, https://pmip3.lsce.ipsl.fr/; Braconnot et al., 2012). A total of 11 scenarios were available as estimates of the mid‐Holocene climate (BCC‐CSM1‐1, CCSM4, CNRM‐CM5, CSIROMk3‐0, CSIRO‐Mk3 l‐1‐2, FGOALS‐g2, GISS‐E2‐R, IPSL‐CM5A‐LR, MIROC‐ESM, MPI‐ESM‐P, and MRICGCM3) and seven scenarios were available for the LGM (CCSM4, CNRMCM5, FGOALS‐g2, IPSL‐CM5A‐LR, MIROC‐ESM, MPI‐ESM‐P, MRI‐CGCM3).

#### 2.8.3 SDM analysis

MAXENT V. 3.4.0 (Phillips et al., 2004; Phillips et al., 2006; Phillips et al., 2009, available through http://www.cs.princeton.edu/schapire/maxent/) was used to assess the potential distribution of *L. strigata*. This is a grid‐based machine‐learning algorithm following the principles of maximum entropy (Jaynes, 1957; Phillips et al., 2004), which derives the potential distribution of a species from presence information compared with a randomly selected set of pseudo‐absences. To assess model performance and to reduce uncertainties, we applied an ensemble modeling approach as suggested by Araújo and New (2007) by computing 100 SDMs each trained with 70% of species presence records and assessed with the remaining 30% through the Area Under the receiver operating Curve (AUC) (Swets, 1988). The value of AUC varies from 0 (low performance) to 1 (perfect discrimination). The average of all models was used for further processing, and results were imported into ArcMap 10 (ESRI, Redlands, CA, USA).

To assess which area exceeds the environmental training conditions under the current and past scenarios, we performed multivariate environmental similarity surfaces (MESS) in MAXENT, which were rescaled to highlight areas of model extrapolations.

## 3 RESULTS

### 3.1 Phylogenetic analyses

Both ML and BI analyses generated similar topologies. Based on the combined genes, the green lizards formed a monophyletic group sister to *Timon,* and the Caspian green lizard was separated from the remaining species with high support values (BS = 100, PP = 1.00) (Figure S1). At the intraspecific level, two distinct clades within *L. strigata* were recovered with high support values (BS = 99%, PP = 1.00). Samples from the eastern distribution formed a separate clade (regional eastern clade), and other individuals from the central and western parts of the distribution range formed another clade (regional western clade) that split into two poorly resolved subclades (a and b) (Figure S1).

Uncorrected genetic distances for Cyt *b* were approximately 2%–3% between individuals from the regional eastern and western clades. Within the regional western clades, the maximum genetic distance among the specimens was about 1% (Table S3).

### 3.2 Estimation of divergence times

**FIGURE 2:**
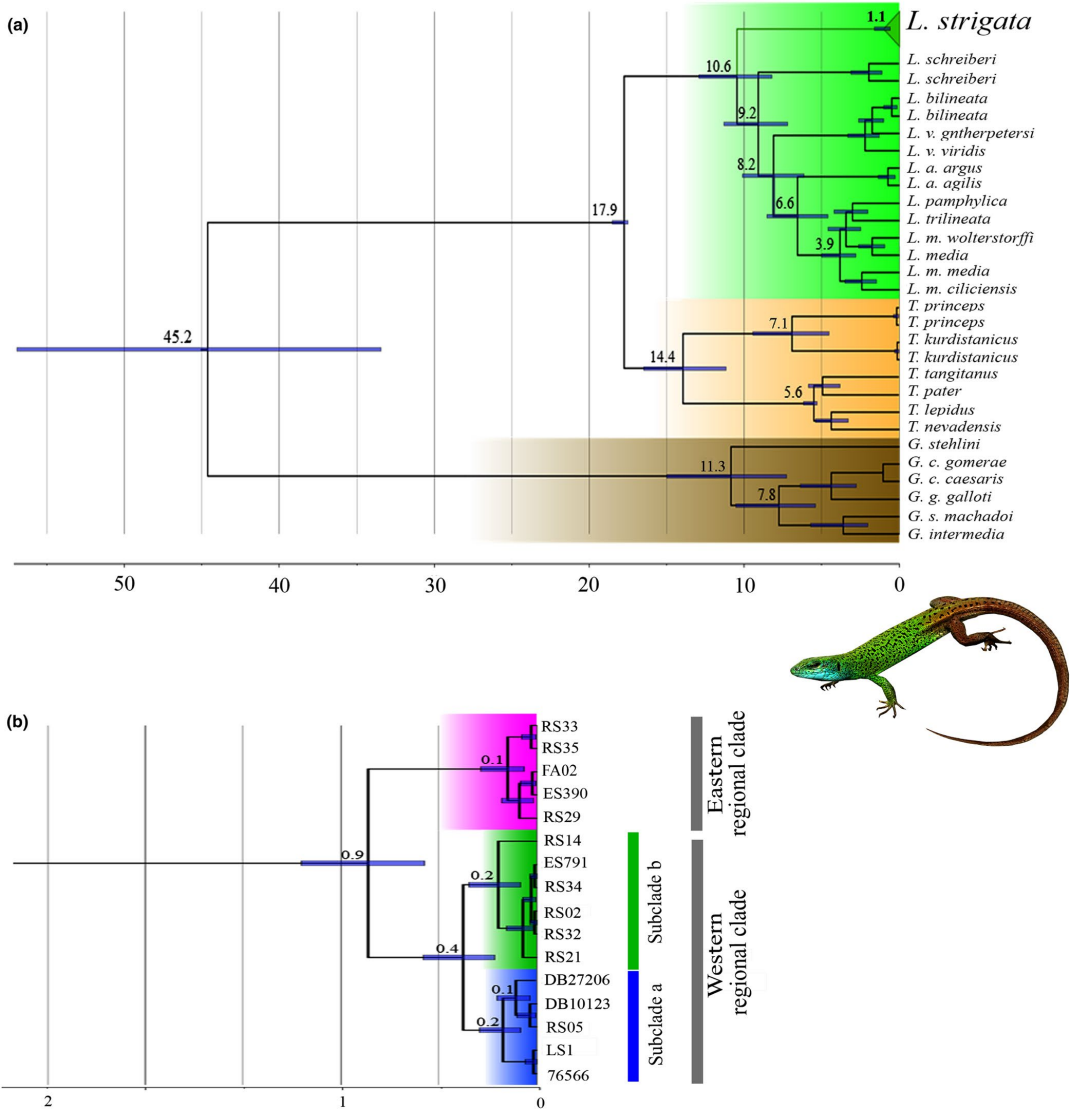
The dated phylogenetic trees using the combined dataset; (a) the time‐calibrated maximum clade credibility tree for the lacertid lizards using the combined dataset; (b) the time‐calibrated maximum clade credibility tree for intraspecific relationships within *Lacerta strigata*. Blue bars show 95% highest posterior density intervals of the estimated node ages; numbers next to the nodes are mean node ages (Mya) (photo by Omid Mozaffari)

Based on the dated tree using the combined dataset, *L. strigata* diverged from the ancestor of other green lizards at 10.6 Mya (95% highest posterior density (henceforth HPD): 8.20–12.93 Mya, Figure 2). The intraspecific divergence between the regional clades within the Caspian green lizard was estimated 1.1 Mya (Dataset 1:95% HPD: 0.57–1.58 Mya). The mutation rates of Cyt *b*, 12S, C‐mos, and β‐fib were estimated 1.47 × 10^–2^, 4.7 × 10^–3^, 7.64 × 10^–4^, and 1.98 × 10^–3^ substitutions per site per million years, respectively. The two main clades within *L. strigata* diverged around 0.9 Mya (Dataset 2:95% HPD: 0.55–1.16 Mya). Within the regional western clade, the two subclades were separated about 0.4 Mya (Dataset 2:95% HPD: 0.21–0.56 Mya).

### 3.3 Population structure

The AMOVA analysis demonstrated that the percentage of variation among populations (eastern and western clades, nearly 85(%)) was higher than within populations (approximately 15(%)), and the *F*st was calculated to be 0.8 (*p* <.05).

### 3.4 Demographic analysis of *Lacerta strigata*

**FIGURE 3:**
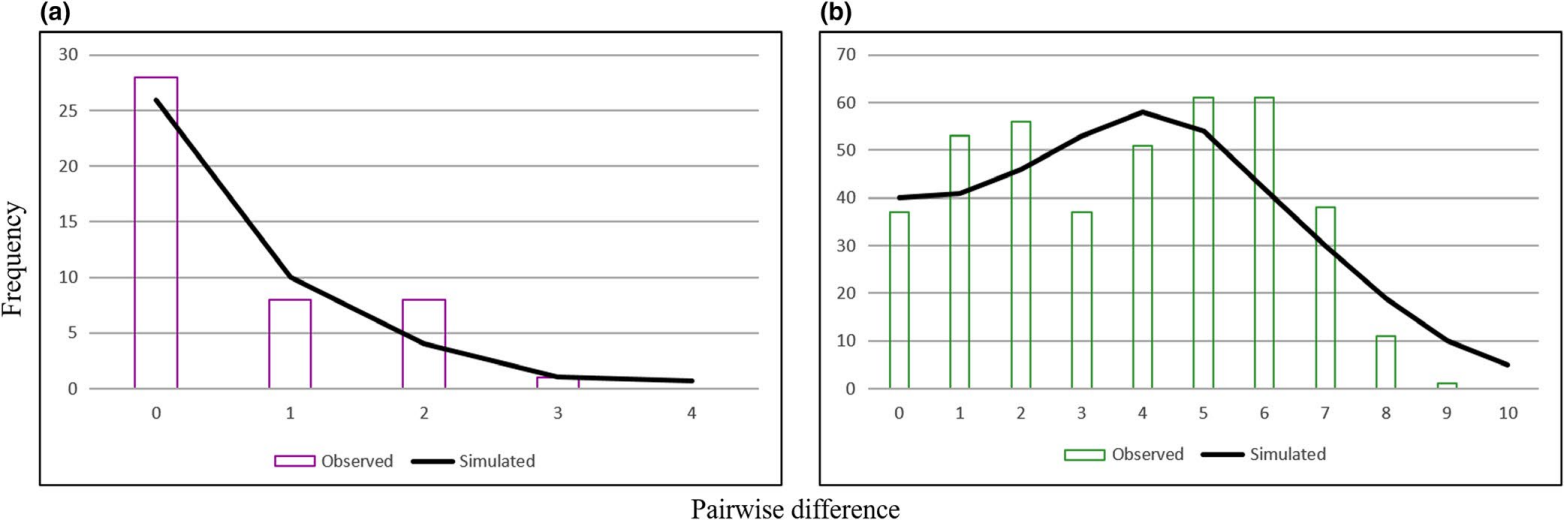
Mismatch distributions of simulated frequencies (line) within the Caspian green lizard compared with the observed frequencies (bar) under the sudden expansion model using Cyt *b*. (a) the MMD diagram for the eastern population shows a recent expansion. (b) the MMD diagram for the western population illustrates a curve with a normal distribution

Hence, the regional eastern and western clades were considered separate populations and analyzed separately. The MMD diagrams for each clade illustrated a unimodal pattern. The regional western clade showed a normal distribution (Figure 3).

Molecular diversity indices evaluated within *L. strigata* and its regional clades are shown in Table 1. The analysis of Fu's *fs* was nonsignificant (*p* >.05) for both populations and Tajima's *D* was significant just for the eastern population (*p* <.05, −1.56).

**TABLE 1:** Molecular diversity indices based on Cyt *b* for *Lacerta strigata* and its regional populations, including the sample size (N), the number of haplotypes (H), haplotype diversity (*h*), nucleotide diversity (π), and the number of polymorphic sites (S)

	N	H	*h*	π	S
*Lacerta strigata*	39	16	0.91	0.012	36
Eastern population	10	3	0.37	0.0007	3
Western population	29	13	0.9	0.004	18

**FIGURE 4:**
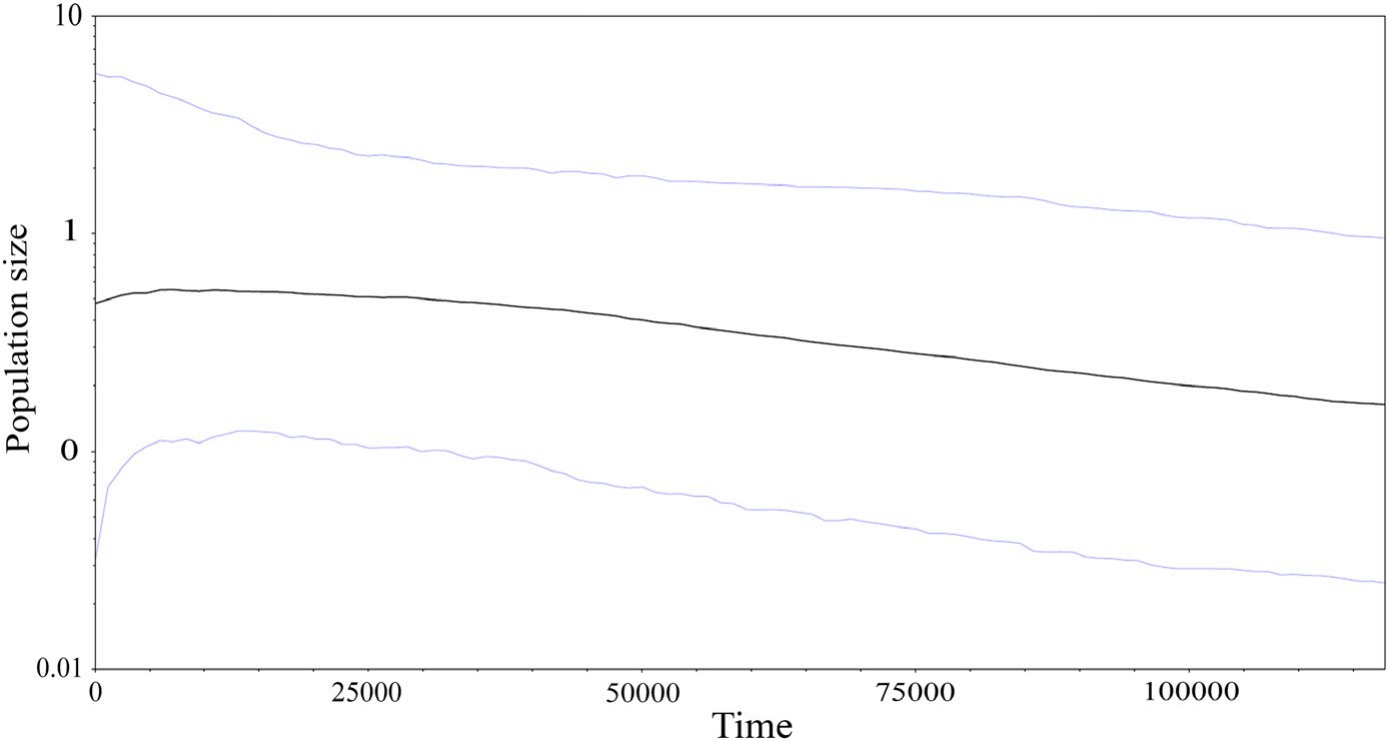
The Bayesian skyline plot showing that the population size changes over time using Cyt *b* for the western regional clad of *Lacerta strigata*. The central line shows the median values of the population size with the 95% highest posterior density intervals

The BSP of the western regional clade (for Cyt *b)* showed a mild increase in *N*_e_ from 120 Kya, with a slight decrease in *N*_e_ toward the present (Figure 4).

### 3.5 Biogeographic analysis

**FIGURE 5:**
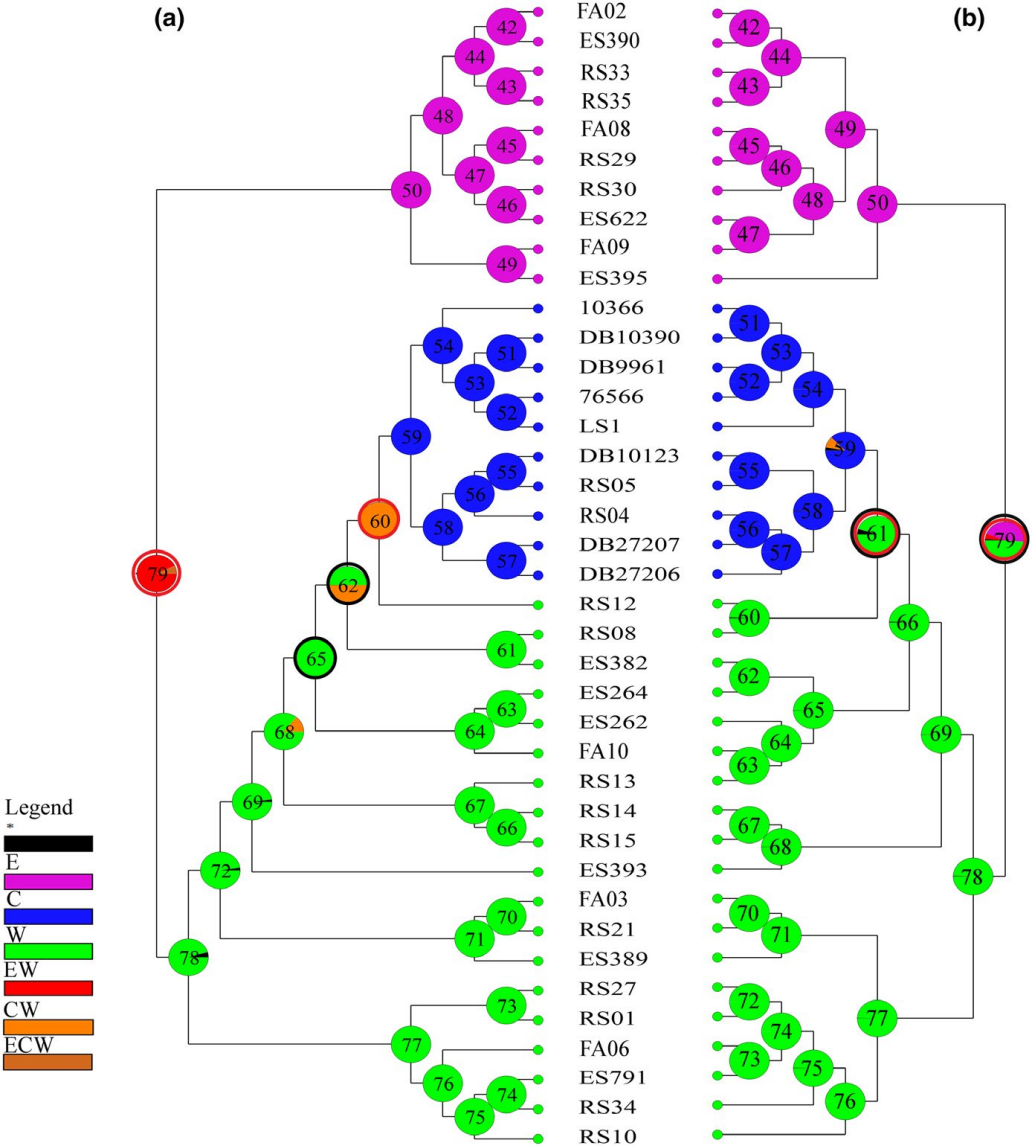
The biogeographic analysis of *Lacerta strigata* using S‐DIVA (a) and BBM (b) based on Cyt *b*. For these analyses, three regions were considered: the eastern distribution (E), the central distribution (C), and the western distribution (W). The black and red circles around the nodes show dispersal and vicariance events, respectively

Based on the current sampling, the S‐DIVA analysis indicated two nodes of dispersal (Node 62 and Node 65) and two nodes of vicariance (Node 60 and Node 79) events. Node 79 is assigned to the divergence of eastern (E) and western (CW) distribution (the MRCA of two regional clades) with Node 60 referred to the divergence of the central and western distribution of *L. strigata*. Node 62 and Node 65 showed dispersal events in the western distribution range of the species (Figure 5a).

The BBM analysis showed two nodes (Node 79 and Node 61) with both dispersal and vicariance events. Based on the analysis, the ancestral Node 79 corresponds to the MRCA of eastern (E) and western (CW) regional clades. The ancestor of the western regional clade (CW) was distributed in the western part of the distribution range (W) (Node 61) (Figure 5b).

### 3.6 Haplotype network

**FIGURE 6:**
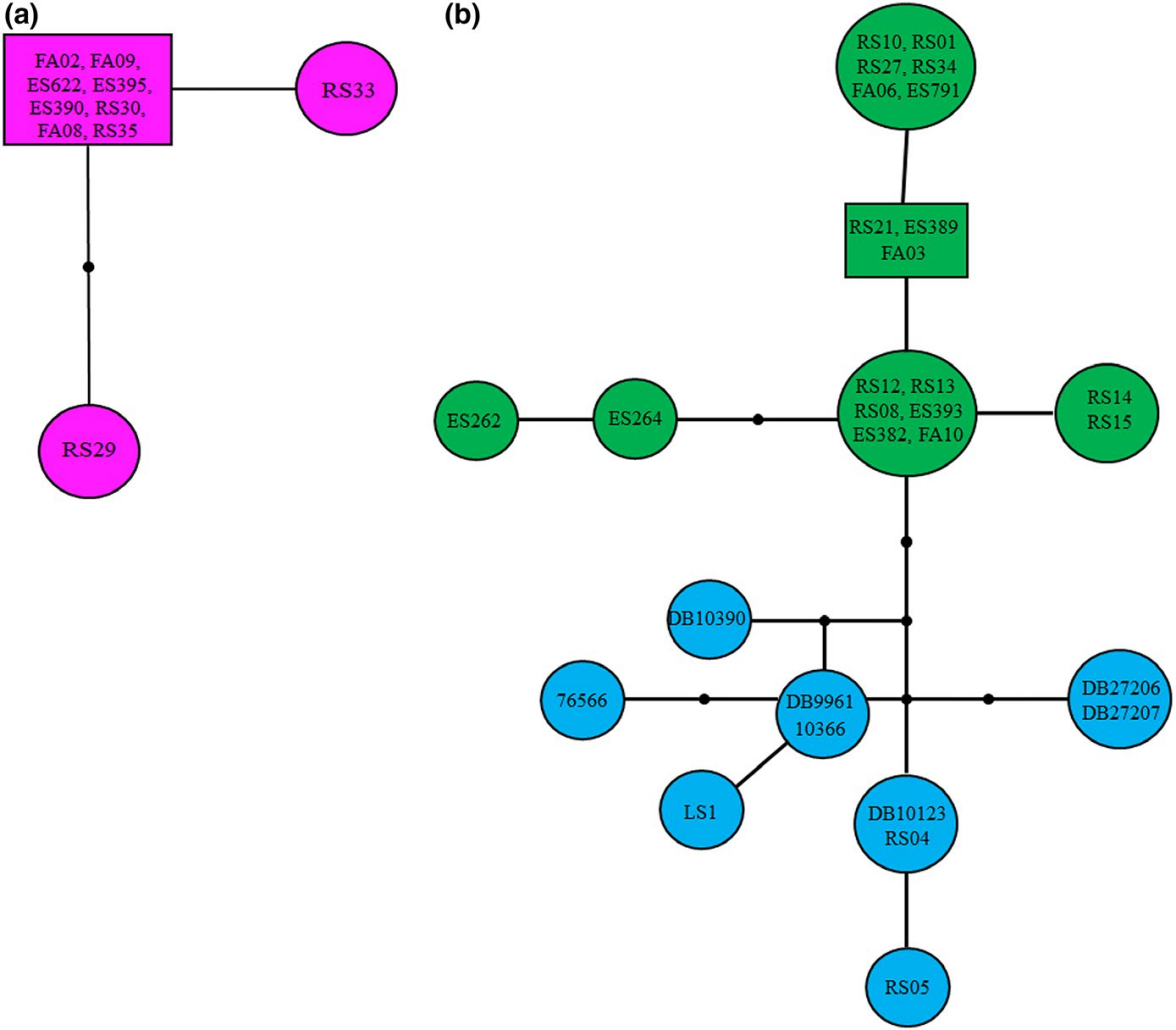
Parsimony haplotype network of the Caspian green lizard using Cyt *b*. The Haplogroup a (pink color) refers to the regional eastern clade, and the Haplogroup b is assigned to the rest of the distribution. The individuals of the subclades a and b are color‐coded with green and blue, respectively (see Figure 2). The squares demonstrate ancestral haplotypes

The parsimony haplotype network showed two haplogroups based on Cyt *b* (Figure 6). Haplogroup a included the eastern samples, and Haplogroup b encompassed the rest (western and central distribution). The ancestral haplotype of Haplogroup b corresponded to RS21 (central Hyrcanian regions) and ES389/FA03 (western Hyrcanian regions) from the central distribution rang. In this haplogroup, two Iranian samples (Astara; including RS04 and RS05) were separated from the other Iranian haplotypes with three‐step mutations. Astara also shared an identical haplotype (RS04) with Nagorno–Karabakh (DB10123) (Figure 6).

### 3.7 Species distribution modeling (past–present)

**FIGURE 7:**
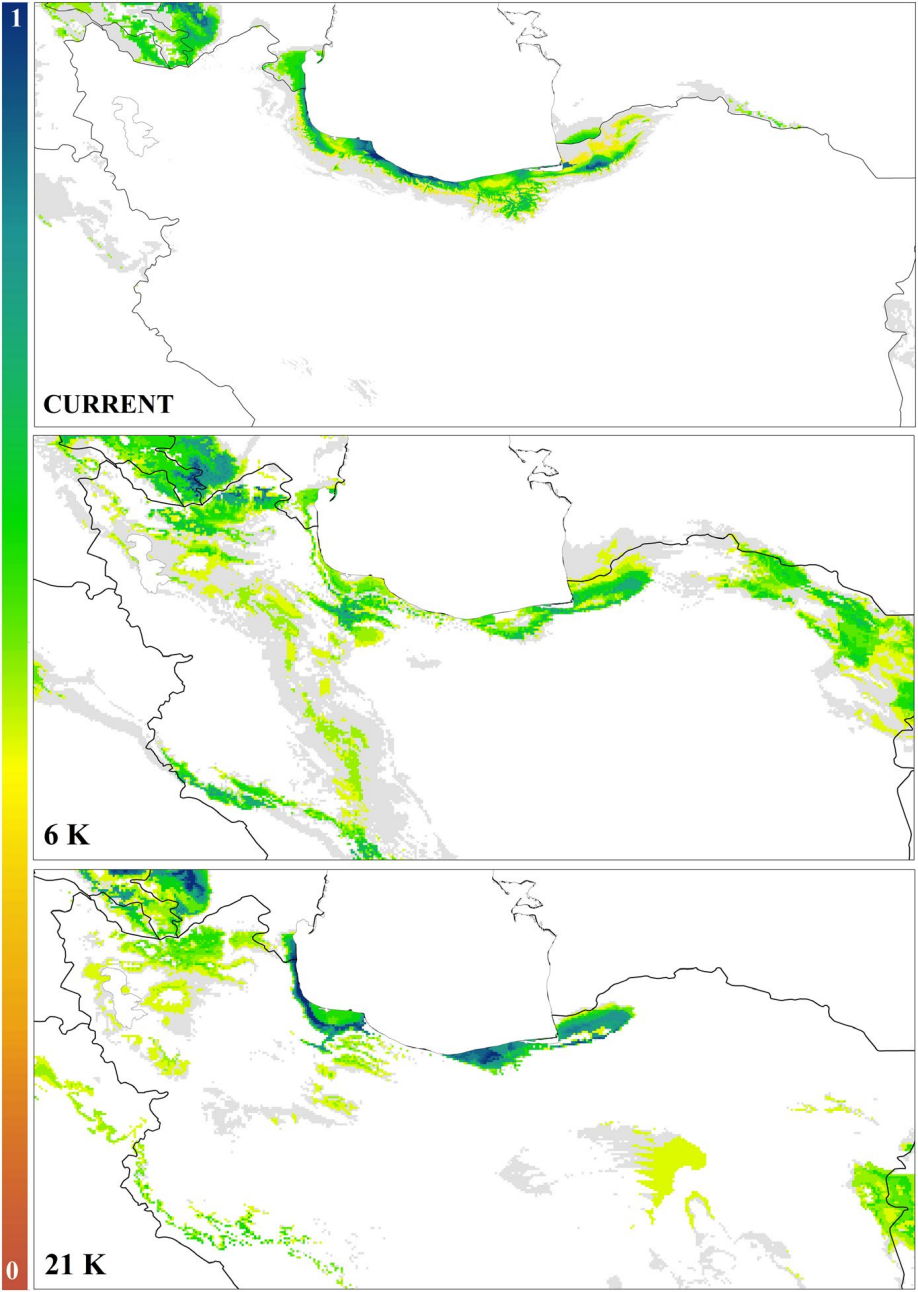
Potential distribution modeling for *Lacerta strigata* under the past (mid‐Holocene (6 kya), the Last Glacial Maximum (LGM; 21 Kya)) and present climatic conditions. The stability of habitat suitability through time indicates potential refugia in this area

Species distribution modeling showed that during the LGM, the habitat suitability for *L. strigata* was contracted into three distinct regions in the lowlands of the Hyrcanian region along the Caspian Sea coasts. The results of comparison with the LGM indicate that during the Mid‐Holocene, suitable habitats gradually increased and the species could the current climate condition, it occurs in a wider distribution range especially in some regions out of the Hyrcanian region in the Caucasus and the southern Alborz (Figure 7).

## 4 DISCUSSION

In this study, we re‐examined the phylogenetic position of the Caspian green lizard among other representatives of the genus and the closest relatives in the family Lacertidae. We also investigated the historical phylogeography of the species to understand the impact of the Pleistocene climatic oscillations on the genetic structure.

### 4.1 Phylogenetic relationships

In the current study, the Caspian green lizard appeared to be the sister species to all other green lizards based on the combined gene dataset (Figure 2 and Figure S1). Although at the generic level, the position of *Lacerta* as sister to *Timon* and the relationships with other genera of Lacertidae has become well established (Ahmadzadeh, Flecks, Rödder, et al., 2013; Godinho et al., 2005), the phylogenetic position of the *L. strigata* within its genus was not well resolved. As such, Godinho et al. (2005) investigated the phylogenetic relationships within green lizards and suggested that *L*. *strigata* (just two individuals from Georgia) was sister to *L. agilis* based on 12S and 16S markers. However, in the same work, *L*. *strigata* was the sister species to all green lizards based on the Cyt *b* gene. Ahmadzadeh, Flecks, Rödder, et al. (2013), using three mtDNA genes (16S, 12S, and Cyt *b*), also suggested *L. strigata* to be a sister clade to *L. viridis* and *L. bilineata*. This uncertainty was not only due to the incomplete taxon sampling but also owing to a different set of markers used in previous studies. Our results revealed that the application of both mtDNA and nuclear markers (multilocus evidence) and more individual samples provide a more robust phylogenetic inference compared with that of the mtDNA.

The intraspecific trees based on the combined dataset supported two main clades within the species, occurring on the eastern and the western parts of the distribution range (see Figure  and Figure S1). The regional eastern clade was limited to the eastern Golestan, whereas the regional western clade included most of the species range from Russia to the western part of the Golestan (Figure 1).

### 4.2 Evolutionary history of *Lacerta strigata*

According to the time‐calibrated tree, the Caspian green lizard was separated from its congeners about 10.6 Mya (95% HPD; 8.20–12.93 Mya) during the Late‐Miocene (Figure 2). This estimated divergence time may appear a bit older than the previous estimation (approximately 9 Mya; Ahmadzadeh, Flecks, Rödder, et al., 2013), which is probably related to the use of nuclear markers and more samples. In the present study, the two main clades were separated in a time period between the Early and Middle Pleistocene (0.9 Mya, 95% HPD: 0.55–1.16 Mya), and the western subclades diverged about 0.4 Mya (95% HPD: 0.17–0.47 Mya). It is comparable to the Hyrcanian wood frog (*Rana pesudodalmatina*) and Fat Dormouse (*Gilis gilis*) that split into the eastern and western lineages about 1.6 Mya (95% HPD: 0.58–2.54 Mya) and 1.19 Mya (95% HPD: 0.55–1.91 Mya), respectively (Ahmadi et al., 2018; N. Amiri, S. Vaissi, F. Aghamir, R. Saberi‐Pirooz, D. Rödder, E. Ebrahimi, & F. Ahmadzadeh, Unpublished data). This pattern of divergence, however, was not observed in the Persian brook salamander (*Paradactylodon persicus*), which is an endemic species from the Hyrcanian Forests (Ahmadzadeh et al., 2020). In contrast, the phylogenetic studies on the greenbelly lizard (*Darevskia chlorogaster*) and Alborz lizard (*Darevskia defilippii*) showed species complexes with deeper phylogenetic separations between the evolutionary lineages in the south of the Caspian Sea (Ahmadzadeh, Flecks, Carretero, Mozaffari, Böhme, et al., 2013).

According to the phylogeographic assessments, we suggest that the climatic oscillations of the Pleistocene were associated with the cladogenesis of *L. strigata*. During the Quaternary stadials, the prevalent climate of the region was cold/dry and the species was likely sheltered in the southern refugia until interstadials when the climate became warmer and moist (Kehl, 2009). The Alborz Mountains and the southern Caspian Sea have been reported as refugia for many other species (Ahmadzadeh, Flecks, Carretero, Mozaffari, Böhme, et al., 2013; Asadi et al., 2019; Saberi‐Pirooz et al., 2018; Veith et al., 2003; Zohary, 1973), acting as sources of subsequent diversifications , which eventually promoted haplotype admixture. Several species in the region showed evidence of shrinks to glacial refugia and subsequent postglacial expansion (Ahmadzadeh, Flecks, Carretero, Mozaffari, Böhme, et al., 2013; Ahmadzadeh et al., 2020).

According to the SDMs, the habitat suitability for the species has been limited to three different areas (western, central, and eastern of the distribution range) in the Hyrcanian Forests since the LGM (Figure 7). Therefore, the regions are identified as Pleistocene refugia because of climate stability in contrast to adjunct regions (like other species in the region; see Ahmadzadeh et al., 2020). Furthermore, the findings of SDMs supported that the range of suitable habitats for the species would have expanded to some regions out of the Hyrcanian region in the Caucasus and the southern Alborz.

In line with the results of the SDMs, the haplotype networks suggest that the regional eastern and western clades presumably possessed separate refugia during the Pleistocene, which may provide evidence for multiple refugia within the main refuge in the southern Caspian Sea. The similar distribution pattern have been also reported for other species (*D. chlorogaster*, *D. deffilipi*, *R. pseudodalmatina*, etc.) occurring in the region which reinforce the hypothesis of multiple refugia (Ahmadzadeh, Flecks, Carretero, Mozaffari, Böhme, et al., 2013; Ahmadzadeh et al., 2020; N. Amiri, S. Vaissi, F. Aghamir, R. Saberi‐Pirooz, D. Rödder, E. Ebrahimi, & F. Ahmadzadeh, Unpublished data). Pleistocene and Holocene fluctuations led to changes in the Caspian Sea level, which affected the vegetation communities of the Hyrcanian Forests. These rapid fluctuations between glacial and interglacial oscillations, with approximately 150 m difference in sea level between the high and low stands (Leroy et al., 2013), are suggested to have contributed to the formation of disconnected refuges within the Hyrcanian Forests. Since the ancestral haplotypes of *L. strigata* were placed in Iran (Figure 6), we hypothesize that other regions (eastern Iran, Russia, Armenia, Azerbaijan, Georgia, and Nagorno‐Karabakh) were colonized from the western part of the Hyrcanian Forests, suggesting a possible postglacial expansion. Indeed, the Astara samples (RS04 and RS05) were placed near the individuals from Nagorno‐Karabakh (DB10123). The Aras valley, making the border between Iran, Azerbaijan, Armenia, Nagorno‐Karabakh, and Turkey, does not seem to act as a geographic barrier for terrestrial vertebrates (Freitas et al., 2016; Saberi‐Pirooz et al., 2018). This region is considered as the area of refuge during cold periods. According to the network analyses, *L. strigata* was present at both riverbanks of the valley with identical haplotypes shared between both sides (RS04 and DB10123).

The S‐DIVA and BBM analyses identified successive dispersal and vicariance events that may have shaped the phylogeographic structure of the species (Figure 5). These events correspond to the divergence of the regional eastern and western clades from the ancestral node. We assume that the shallow divergence of the genetic lineages may have resulted from short‐distance dispersal (Irwin, 2002). Also, it is suggested that dispersal to other regions during the postglacial and the repeated sequence of restriction and expansion in the Hyrcanian Forests led to the allopatric isolation of forest‐dwelling species (Ahmadi et al., 2018).

### 4.3 Genetic structure and demography

Based on the AMOVA analysis, the two main clades (regional eastern and western clades) were considered as distinct populations (*F*st = 0.8). Moreover, the MMD diagrams and the demographic analysis (Tajima's *D* and Fu's *fs*) revealed a recent expansion of the regional eastern clade (Figure 3). The nonsignificant and negative values for the demographic analysis of the regional western clade did not allow for any inference on the historical demography (see Parvizi et al., 2018).

Overall, the patterns of haplotype network, as well as the neutrality statistics and MMD results, were generally consistent with the hypothesis of a recent expansion of the species. The BSP displayed a mild increase in *N*_e_ for the regional western clade from about 120 Kya, suggesting that the species responded favorably to the past environment of the refugia during the Late‐Pleistocene. Besides, these refugia may have buffered unfavorable climatic conditions during glacial cycles of the Late‐Pleistocene, which promoted the *N_e_*.

The haplotype and genetic diversity indices were high within the species (*h* = 0.91, *π* = 0.012). These indices were low for the regional eastern clade compared with the regional western clade (Table 1). We suggest that the weak genetic structure (*h* and *π*) of the eastern population is probably due to a population expansion from a small number of founder individuals in the eastern part of the Hyrcanian Forests (also see Ahmadi et al., 2018).

In general, it seems that the genetic structure of the species may have potentially developed through the isolation of refugial populations in two separate areas. It is supposed that during the climatic oscillations, subsequent local contraction, and expansion events probably shaped the current species distribution range, though there was no plenty of time for a more pronounced genetic structure.

## 5 CONCLUSION

In the present study, the phylogenetic position of *L. strigata* as the sister taxon to other congeneric species was well supported. The phylogenetic analyses supported two intraspecific main clades (eastern and western), with the regional western clade that split into two subclades. The time‐calibrated analysis showed that the intraspecific divergence for the main clades took place in a time period between the Early and Middle Pleistocene, while the regional western subclades were separated during the Middle Pleistocene. The demographic inference suggested that *L. strigata* experienced a mild population expansion coinciding with the Late‐Pleistocene climate oscillations. As no natural barriers are recognized for the separation of such clades, we assume that short‐distance dispersal and isolation in several hidden refugia were probably the drivers of the genetic structure. These aspects have to be further confirmed using detailed population genetics and more samples.

## CONFLICT OF INTEREST

None declared.

## AUTHOR CONTRIBUTIONS

**Reihaneh Saberi‐Pirooz:** Conceptualization (supporting); formal analysis (lead); methodology (lead); project administration (equal); software (lead); visualization (supporting); writing–original draft (lead). **Hassan Rajabi** **‐** **Maham**: Funding acquisition (equal); Data curation (equal); formal analysis (equal); supervision (supporting); writing–review and editing (equal). **Faraham Ahmadzadeh:** Funding acquisition (equal); Conceptualization (lead); data curation (lead); formal analysis (equal); methodology (equal); supervision (lead); writing–original draft (equal); writing–review and editing (equal). **Bahram H. Kiabi:** Conceptualization (equal); supervision (equal); writing–review and editing (equal). **Mohammad Javidkar:** Data curation (equal); formal analysis (supporting); methodology (supporting); software (supporting); writing–original draft (equal); writing–review and editing (supporting). **Miguel A. Carretero:** Funding acquisition (equal); investigation (equal); writing–review and editing (equal).

## Supporting information

Supplementary Material

Table S4

## Data Availability

The data for this study, including accession numbers for genetic sequences deposited on NCBI GenBank, are recorded in the appendix (Table S1).
