# Supplementary material for: Pleistocene climate fluctuations as the major driver of genetic diversity and distribution patterns of the Caspian green lizard, Lacerta strigata Eichwald, 1831
Source: Ecol Evol. 2021 May 2;11(11):6927–40. doi: 10.1002/ece3.7543 (PMC8207146; doi:10.1002/ece3.7543)

**SUPPORTING INFORMATION**

Pleistocene climate fluctuations as the major driver of genetic diversity and distribution patterns of the Caspian green lizard, *Lacerta strigata* Eichwald, 1831

Reihaneh Saberi-Pirooz, Hassan Rajabi-Maham, Faraham Ahmadzadeh, Bahram Kiabi, Mohammad Javidkar, Miguel A. Carretero

Table S1. The data set of *Lacerta strigata* used in this study includes code ID, locality, coordinates, and accession numbers for Cyt *b*, 12S, C-mos, and β-fib genes

| Code ID | Location | Longitude | Latitude | | Accession numbers | | | | | | |
| --- | --- | --- | --- | --- | --- | --- | --- | --- | --- | --- | --- |
|  |  |  |  |  | Cyt *b* | 12S | | C-mos | | β-fib | |
| DB10390 | Armenia | 40.75483 | 44.88509 | | MH974527 | _ | | _ | | _ | |
| DB9961 | Armenia | 40.6095 | 44.60896 | | MH974530 | _ | | _ | | _ | |
| 10366 | Armenia | 40.38530167 | 45.194055 | | MW592672 | _ | | _ | | _ | |
| DB10123 | Nagorno-Karabakh | 40.13091 | 46.46458 | | MH974520 | MW589401 | | _ | | MW591731 | |
| 76566 | Georgia | 41.59147 | 44.67063 | | MH974535 | MW589393 | | MW591709 | | MW591723 | |
| LS1 | Georgia | 41.79734 | 45.76174 | | MH974536 | _ | | _ | | _ | |
| DB27206 | Russia | 44.02361 | 43.09222 | | MW592673 | MW589402 | | MW591717 | | MW591732 | |
| DB27207 | Russia | 44.69139 | 44.17444 | | MW592674 | _ | | _ | | _ | |
| RS14 | Iran, Ardabil province | 38.61242742 | 48.44288 | | MH974524 | MW589394 | | MW591710 | | MW591724 | |
| RS15 | Iran, Ardabil province | 38.61242742 | 48.44288 | | MH974525 | _ | | _ | | _ | |
| RS10 (RS02) | Iran, Gilan province | 37.4649 | 49.93769 | | MH974521 | MW589397 | | MW591713 | | MW591727 | |
| RS12 | Iran, Gilan province | 37.1422 | 50.06072 | | MH974522 | _ | | _ | | _ | |
| RS13 | Iran, Gilan province | 37.12944 | 50.22565 | | MH974523 | _ | | _ | | _ | |
| RS05 | Iran, Gilan province | 38.42997 | 48.88097 | | MH974533 | MW589395 | | MW591711 | | MW591725 | |
| RS08 | Iran, Gilan province | 37.23135 | 49.31835 | | MH974537 | _ | | _ | | _ | |
| FA10 | Iran, Gilan province | 37.016045 | 49.85139 | | MH974539 | _ | | _ | | _ | |
| RS04 | Iran, Gilan province | 38.42997 | 48.88097 | | MW592675 | _ | | _ | | _ | |
| ES264 | Iran, Gilan province | 37.74594 | 48.93964 | | MW592676 | _ | | _ | | _ | |
| ES262 | Iran, Gilan province | 37.74594 | 48.93964 | | MW592677 | _ | | _ | | _ | |
| ES382 | Iran, Gilan province | 36.93757 | 50.07496 | | MW592678 | _ | | _ | | _ | |
| ES393 | Iran, Gilan province | 36.93757 | 50.07496 | | MW592679 | _ | | _ | | _ | |
| RS33 | Iran, Golestan province | 37.48682 | 55.50404 | | MH974531 | MW589406 | | MW591721 | | MW591736 | |
| RS34 | Iran, Golestan province | 36.81629 | 54.04977 | | MH974532 | MW589399 | | MW591715 | | MW591729 | |
| FA02 | Iran, Golestan province | 37.13716 | 54.61338 | | MH974538 | MW589405 | | MW591720 | | MW591735 | |
| FA03 | Iran, Golestan province | 37.42811 | 54.63877 | | MW592680 | _ | | _ | | _ | |
| FA09 | Iran, Golestan province | 36.9837 | 54.85492 | | MW592681 | _ | | _ | | _ | |
| ES622 | Iran, Golestan province | 37.217889 | 55.26108 | | MW592682 | _ | | _ | | _ | |
| FA08 | Iran, Golestan province | 37.13524 | 54.65492 | | MW592683 | _ | | _ | | _ | |
| ES390 | Iran, Golestan province | 37.062 | 54.69099 | | MW592684 | MW589403 | | MW591718 | | MW591733 | |
| ES395 | Iran, Golestan province | 37.062 | 54.69099 | | MW592685 | _ | | _ | | _ | |
| ES791 | Iran, Golestan province | 37.13509 | 54.60664 | | MW592686 | MW589396 | | MW591712 | | MW591726 | |
| ES389 | Iran, Golestan province | 37.01366 | 54.52779 | | MW592687 | _ | | _ | | _ | |
| RS29 | Iran, Golestan province | 37.1986553 | 54.72198 | | MW592688 | MW589404 | | MW591719 | | MW591734 | |
| RS30 | Iran, Golestan province | 37.1986553 | 54.72198 | | MW592689 | _ | | _ | | _ | |
| RS35 | Iran, Golestan province | 37.48682 | 55.50404 | | MW592690 | MW589407 | | MW591722 | | MW591737 | |
| RS01 | Iran, Mazandaran province | 36.6613611 | 52.53141 | | MH974526 | _ | | _ | | _ | |
| RS21 | Iran, Mazandaran province | 36.65168 | 51.50098 | | MH974528 | MW589400 | | MW591716 | | MW591730 | |
| RS32 | Iran, Mazandaran province | 36.5478 | 53.1321 | | MH974529 | MW589398 | | MW591714 | | MW591728 | |
| FA06 | Iran, Mazandaran province | 36.531424 | 53.18641 | MH974534 | | | _ | | _ | | _ |

Table S2. The list of Lacertidae species used for the phylogenetic analyses including country, and accession numbers for Cyt *b*, 12S, C-mos, and β-fib.

| Species | Country | Accession numbers | | | |
| --- | --- | --- | --- | --- | --- |
|  |  | Cyt *b* | 12S | C-mos | β-fib |
| *Gallotia caesaris caesaris* | Spain | AY151843 | AY151922 | AY152006 |  |
| *Gallotia caesaris gomerae* | Spain | AY151842 | AY151921 | AY152005 | _ |
| *Gallotia galloti galloti* | Spain | AY151840 | AY151919 | AY152003 |  |
| *Gallotia intermedia* | Spain | AY151844 | AY151923 | AY152007 | _ |
| *Gallotia simonyi machadoi* | Spain | AF101219 | AY151924 | AY152008 |  |
| *Gallotia stehlini* | Spain | AY151838 | AY151917 | AY152001 | _ |
| *Lacerta agilis argus* | Austria | DQ097090 | AF149947 | DQ097136 | DQ097108 |
| *Lacerta agilis agilis* | Netherlands | AF080299 | DQ097096 | AF315397 | DQ097109 |
| *Lacerta bilineata* | Spain | _ | AF149955 | DQ097134 | DQ097116 |
| *Lacerta bilineata* | Italy | DQ097087 | AF149956 | DQ097135 | _ |
| *Lacerta media ciliciensis* | Turkey | KC896972 | KC896860 | _ | _ |
| *Lacerta media media* | Iran | KC896983 | KC896871 | _ | _ |
| *Lacerta media* | _ | KC896975 | KC896863 | _ | _ |
| *Lacerta media wolterstorffi* | Lebanon | KC897008 | KC896896 | DQ097144 | DQ097105 |
| *Lacerta pamphylica* | Turkey | DQ097089 | AF149954 | DQ097142 | DQ097103 |
| *Lacerta schreiberi* | Portugal | AF386785 | AF206591 | DQ097127 | _ |
| *Lacerta schreiberi* | Spain | AF386784 | DQ097093 | DQ097126 | DQ097101 |
| *Lacerta strigata* | Georgia | DQ097091 | DQ097094 | DQ097137 | DQ097107 |
| *Lacerta trilineata polylepidota* | Turkey | DQ097092 | AF149948 | DQ097138 | DQ097111 |
| *Lacerta viridis viridis* | Greece | AF233423 | AF149958 | DQ097132 | DQ097122 |
| *Lacerta viridis gntherpetersi* | Greece | AF233424 | AF149959 | DQ097131 | DQ097119 |
| *Timon kurdistanicus* | Iran | JQ425831 | JQ425791 | JQ425828 | JQ425824 |
| *Timon kurdistanicus* | Iran | JQ425835 | JQ425795 | JQ425829 | JQ425825 |
| *Timon princeps* | Iran | JQ425839 | JQ425799 | JQ425826 | JQ425822 |
| *Timon princeps* | Iran | JQ425843 | JQ425803 | JQ425827 | JQ425823 |
| *Timon pater* | Tunisia | AF378967 | AF378947 | EU365409 | EU365413 |
| *Timon tangitanus* | Morocco | AF378961 | AF378945 | EU365410 | EU365419 |
| *Timon nevadensis* | Spain | AF379010 | AF378941 | EU365408 | EU365421 |
| *Timon lepidus lepidus* | Portugal | AF378970 | AF378942 | EU365407 | EU365428 |


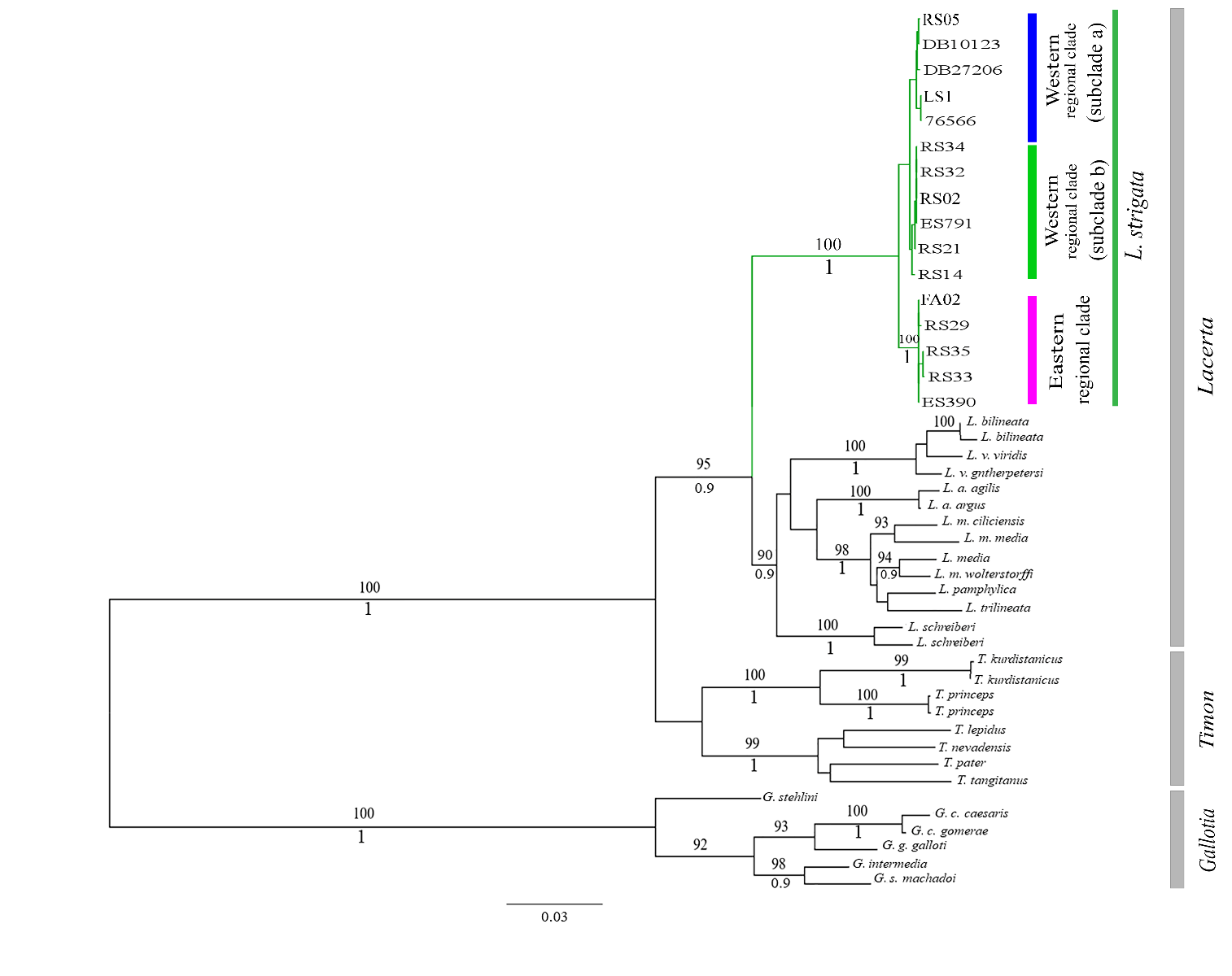
­­

Figure S1. The ML phylogenetic tree of the lacertid lizards using the combined genes (Cyt *b*, 12S, C-mos, and β-fib; 2178 bp). The topologies of BI and ML trees are the same, therefore the ML tree is shown. The values above and below the branches show bootstrap supports for the ML and posterior probabilities for the BI, respectively.

Table S3: Uncorrected genetic *p*-distances within *L. strigata* using Cyt *b*. These *p*-distances show the amount of divergence between the individuals of *L. strigata* (see voucher codes in Table S1)


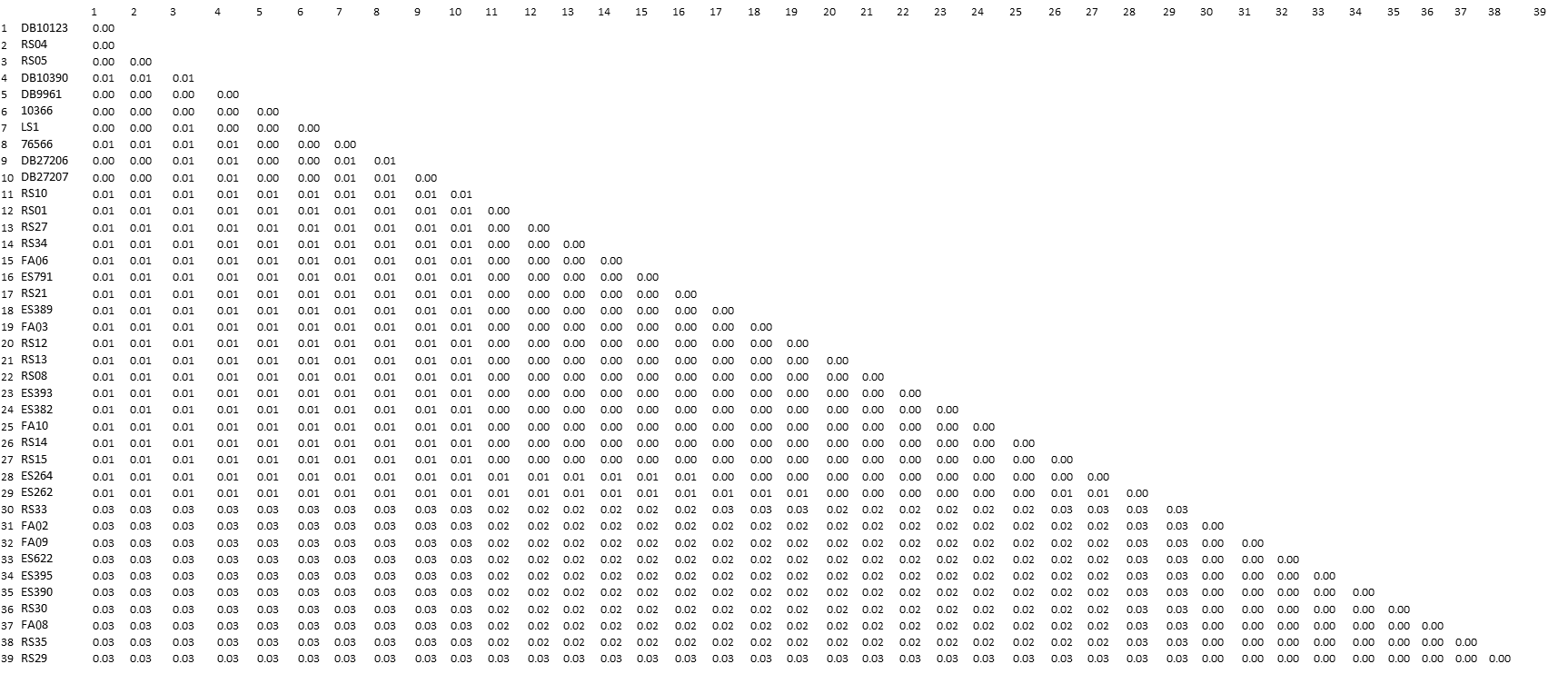

Supplement: Supplementary file 1 — Supplementary Material [file ECE3-11-6927-s001.docx]
